# What is culturally informed psychiatry? Cultural understanding and withdrawal in the clinical encounter

**DOI:** 10.1192/pb.bp.114.047936

**Published:** 2015-08

**Authors:** Anne Birgitte Leseth

**Affiliations:** 1 Oslo and Akershus University College of Applied Sciences, Norway

## Abstract

What is culturally informed psychiatry? What does it mean, and why is it important? These questions are discussed with a focus on the cultural aspects of the clinical encounter. The DSM-5 *Outline for Cultural Formulation* was developed as a method of assessing the cultural factors affecting the clinical encounter. It calls for the assessment of the cultural features of the relationship between the patient and the clinician; however, there is a lack of debate about what this means in practice. Clinicians run the risk of withdrawal rather than cultural understanding when facing patients with different cultural backgrounds. Using ethnographic material from anthropological fieldwork, I suggest that the encounter with cultural differences could be a useful point of departure for the clinician to develop cultural understanding. It is argued that recognising the experiences of differences is crucial in strengthening transcultural communication and preventing misdiagnosis in the clinician–patient encounter.

‘Pathology’ is always a measure of difference.^1^

Increased evidence points towards culture as one aspect influencing the prognosis, cause, manifestation and course of mental health problems. Mental health problems are more frequently misdiagnosed among patients from ethnic minority, immigrant and refugee groups than among native-born patients.^2^ Misdiagnoses may involve failing to recognise the mental health condition or mistaking culturally normative behaviour for psychopathology.^3^ The cultural formulation in DSM-5 was developed as a standard method of applying cultural perspectives to the clinical evaluation.^4^ The *Outline for Cultural Formation* (OCF) has been field-tested for diagnostic usefulness among clinicians.^5^ The cultural formulation has been praised as the most outstanding anthropological contribution to psychiatry, yet it has also been met by critiques by some medical anthropologists and psychiatrists. With the cultural formulation in the DSM-5 and its critique as the point of departure, this article questions what culturally informed psychiatry means. The argument is that the encounter between the patient and the clinician is a crucial, yet underappreciated, position from where the clinician might develop a cultural understanding.

## DSM and the problem with culture

The contemporary model of the cultural formulation in the DSM-5 and DSM-IV dates from the criticism of the insensitivity to cultural issues in the DSM-III.^6^ The institutional culture of medicine was characterised as a ‘culture of no culture’, with limited interest in cultural issues.^7^ The OCF was first published in the DSM-IV in 1984, with a glossary of culture-bound syndromes, culturally relevant diagnostic categories and cultural considerations in the narratives introducing each chapter.^8^ The National Institute of Mental Health in the USA supported the creation of a culture and diagnosis group in 1991, whose main goal was to advise the DSM-IV task force on how to make culture more central to the manual.^9^ However, although the culture and diagnosis group succeeded in emphasising the relevance of cultural issues in psychiatry, there has been criticism of the cultural formulation from leading medical anthropologists over the years. This criticism can be summed up in the following four points.

1. There is a lack of evidence that culturally informed therapeutic practices work.^10,11^ Kleinman & Benson suggest that the major claims about the value of cultural competence for professional caregiving are not supported by evaluation research showing that systematic attention to culture really improves clinical services.^11^ This lack of evidence is a failure of outcome research to take culture seriously enough to asses the cost-effectiveness of culturally informed therapeutic practices.^11^ Moreover, evidence-based practice should include a broader view of evidence that takes into account how different cultures recognise different ways of knowing.^10^
2. In striking contrast to its current use in anthropology, the concept of culture tends to be defined in medicine as something possessed by the patient and not the doctor.
3. Culture is conceived as a confounding variable that White practitioners must deal with when they interact with people from ethnic minority groups.^1^ The locus of normality is white. Taking whiteness for granted represents a specific view of culture that is shared by many White practitioners.^13^ The Black and minority ethnic patient is construed as the object of specialised knowledge, while the professional and their cultural context are left unquestioned.^13^ Historically, this view is reflected in the ways of linking psychiatric knowledge production and implementation to a strictly Western agency, while non-Westerners are often posited as passive receivers of this knowledge.^14^
4. Clinicians are criticised for lacking a cultural self-reflexive attitude towards their own professional practice.^15^

The cultural formulation of the DSM-5 aims to address these criticisms. The OCF is converted into the Cultural Formulation Interview (CFI), including 16 questions focusing on the patient's presenting concerns and definition of their problem and idiom of distress.^16(p.749–760)^ Culture is defined as the systems of knowledge, concepts, rules and practices that are learned and transmitted across generations, yet are open, dynamic and undergo continuous changes over time.

The formulation emphasises that cultural information must not be overgeneralised or stereotype groups of fixed cultural traits.^16(p.749)^ The OCF calls for a systematic assessment of the following four categories when conducting the CFI:

- cultural identity of the individual
- cultural conceptualisation of distress
- psychosocial stressors and cultural features of vulnerability and resilience
- cultural features of the relationship between the individual and the clinician.^16(p.750)^

A persistent challenge faced by clinicians in implementing the cultural formulation in the DSM-5 is how to translate insights from the social sciences in intelligible, practical and sustainable ways.^17^

I aim to delve deeper into the fourth category: the clinical relationship. I aim to translate insights from anthropological fieldwork into practical methods for clinicians. Cultural difference can affect the clinical encounter, and part of the role of culturally informed psychiatry is to address this difference. Using ethnographic material from my anthropological fieldwork in Tanzania, I explore the cultural experience evoked in the encounter. This knowledge is transferred into the context of medical practice.

## White person, where are you going?

Dressed in training tights, a t-shirt and running shoes, I stood in an outside gathering hall in a squatter area of Dar es Salaam, Tanzania, in January 1989. I was preparing to exercise young Tanzanian women in aerobic. As a new teacher in physical education from Norway, I was engaged for 1 month in the Norwegian sports development project, Sports for All, with the aim of giving working class women the opportunity to exercise. Twenty women arrived; none of them was wearing training gear or running shoes, but they had colourful dresses or blouses and skirts and were barefoot. I started to count one, two, three, and then pressed the play button on the music player. The Tanzanian women questioned every move I made. They wondered if they would get paid for exercising. They wanted to touch my white, pale skin and glanced at my freckles. They laughed in a friendly way at jumping up and down, commenting that the moves were a bit childish. Every day, when I left the daily training, people glanced at me and called out: ‘White person, where are you going?’ This question persecuted me in the years to come during several field visits to Tanzania and filled me with uneasiness, curiosity and amazement.^18^

The amazement was first and foremost a ‘culture shock’ that caused me to question my whiteness, my way of practice and instruction. The Danish anthropologist Kirsten Hastrup used the term ‘amazement’ as a cultural pivotal point and a way of understanding that brings a person through emotional and embodied states.^19^ When our habitual practices are questioned we become amazed. When the Tanzanian women questioned the way I moved my arms up and down, they questioned body practices that were part of my professional education and that I had taken for granted. When they commented on my moves or skin colour, I was amazed, as I never thought about these in terms other than ‘normal ways’. The amazement is embodied and cultured.^20^

It was in the encounter with the Tanzanian women that I became aware of my habitual practices and cultural ways. It was by being amazed that I became conscious that, like the Tanzanians, I too possessed systems of knowledge, concepts, rules and practices that are learned and transmitted across generations, yet are open, dynamic and undergo continuous changes over time.

Next, I will discuss how this experience can be relevant to a Western clinician by elaborating on some characteristics of the encounter between the doctor and patient, as discussed by Roland Littlewood, among others.^21^

## The clinical encounter

The encounter between the psychiatrist and the patient involves two people who have their own expectations. If the doctor–patient situation is familiar to both, they will each probably make an effort to live up to the other's expectations. For example, the expectation that the patient is seeking advice to solve a specific problem and that the doctor is an expert who will provide this advice. However, the psychiatrist and the patient face challenges if their cultural backgrounds differ considerably. The psychiatrist might have a less clear expectation of how the patient is likely to behave and what the limits of normality and abnormality are. In this sense, the encounter between the psychiatrist and the patient shares several similarities with the encounter between the researcher and informants from different cultural contexts. The psychiatrist's attitude towards a patient from a minority ethnic background will be informed by the clinician's own experiences, stereotypes and conscious and unconscious racial assumptions. For example, the clinician might have certain race-related assumptions and the patient might be assumed to have a core set of beliefs.^22^ Stereotypes of how other groups of people (such as Danish-Somalis, Native Americans and British Pakistanis) tend to behave influence the treatment options. Patients have their own expectations and the extent to which they see themselves as mentally ill varies with cultural background. What might be tolerated in Tanzania, such as spirit possession, witchcraft and healing ritual, are regarded as forms of abnormality (if not mental illness), in Britain.^23^ Patients with a migrant background seeking help in psychiatric out-patient clinics in European counties might have experiences of mental illness that differ from the doctor's experiences. For example, they might experience their illness as a physical disability or have felt lost in a fragmented health system.^5^ How the psychiatrist copes with their own amazement is therefore of importance.^24^ I present two cases based on my own experience to demonstrate two ways clinicians might respond to amazement.

## Amazement: cultural understanding or withdrawal?

Culturally informed psychiatry is required in domestic contexts familiar to the clinician. Clinicians' experiences of differences, such as language barriers, patients' expressions of distress and orientations of belief can trigger fear, anxiety and amazement. To be aware of one's own amazement might uncover issues taken for granted that the clinician assumes to be inevitable and universal. These often unnoticed assumptions may refer to all sorts of beliefs, habits, practices and values, from body comportment to being accustomed to urban infrastructure. I suggest two responses to this amazement: cultural understanding and withdrawal.

### Example 1

At a Swedish conference on psychiatry and the cultural formulation in the DSM-5, the participants discussed diagnostic practices for asylum seekers. A psychiatrist said: ‘It is very hard when you receive a refugee. The first thing you are supposed to do is to consider the person's mental health. I was really amazed when I discovered that my patient's strong sense of confusion was not necessarily due to his mental condition. Rather, it could be ascribed to his overwhelmed experience of seeing a Swedish city … We should not be too quick to diagnose refugees with post-traumatic stress disorder [PTSD], before they have time to get used to the new cultural context.’ The discussion that followed concerned various clinical experiences with ‘cultural differences’, such as the difficulty in understanding patients' expressions of distress and in making meaningful diagnostic evaluations.^5^ The psychiatrist was amazed at his own cultural attention to the patient. He took for granted, as a prerequisite for professional practice, that the patient was familiar with the material surroundings of the therapy practice: the buildings, roads, transport and so on. In the encounter with the patient, he had quickly diagnosed the patient's confusedness as PTSD. Although the refugee suffered in some ways because of his experience in a war-ridden country, the psychiatrist's amazement and reflection on it gave him alternative ways of interpreting the patient's suffering.

The psychiatrist was able to reflect critically upon his own cultural background and his taken-for-granted perspectives, which places him in a better position to understand and reconsider the mental state of the asylum seeker.

### Example 2

A Norwegian family therapist was observing an 8-year-old Afghan boy to assess his mental health. The boy was not very talkative and was by himself. He had arrived in Norway with his family some years ago. His parents were reluctant towards family therapy as it appeared quite unfamiliar to them. The therapist, on the other hand, aimed at making a decision on the diagnosis of the boy. The parents came with the boy the day that the therapist informed them about the boy's problems. She explained to them that their son was diagnosed with autism spectrum disorder.^16(p.50–55)^ The therapist informed the parents about their welfare rights that followed their son's acknowledged diagnosis, information that was quite new to them. The therapist made a new appointment with the parents and their son. However, only the father attended the next appointment. He informed the therapist that they had received a letter from the director of the hospital claiming the director regretted that their son received the diagnosis and confirmed its withdrawal. Their son did not have autism. The father said there was no more to do, and politely left. Leaving behind the astonished therapist, the director of the hospital confirmed that the letter was never written. When the therapist called the father of the boy to tell him that there was never such a letter, the father replied that it must have been lost. The therapist was never in contact with the family again.

When giving this account, the therapist expressed great frustration that the family did not see the value of this diagnosis. She saw it as her duty to assess and diagnose the patient. She was amazed that the family did not accept the diagnosis and she thought of this as a cultural problem. Therefore, she did not try to explore how her own amazement could be interpreted as a cultural response to an unfamiliar situation (that the parents did not accept the diagnosis). In the encounter, the family therapist took it for granted that the patient and their relatives would accept the diagnosis given to them.

The amazed clinicians might not be able to develop an understanding in the encounter with the patient, but might withdraw instead. Clinicians may respond to their own experience of difference by using cultural categories on the patients, setting themselves as the normative standard. Imposing identities on patients, such as ‘boy with autism’, ‘woman with bipolar disorder’, ‘man with suicidal tendencies’, helps clinicians feel more secure with their own identity and withdraw from an alternative identity experienced by the patient.^25^

To be able to learn from one's own amazement to develop understanding as a researcher requires a culturally reflexive research position. In anthropological fieldwork the researcher must take into account that they are always part of the situation being studied. Part of the criticism of the cultural formulation, as discussed earlier, is a lack of self-reflexivity among clinicians when it comes to their professional background. A clinician who understands something of their own cultural background and how it contributes to their values, perceptions and personal style is in a better position to learn from the clinical encounter with others.^26^ Amazement that stems from the encounter with difference is, in this regard, a trigger point.

The two examples above demonstrate that it is not a straightforward matter for the clinician to use amazement as a tool to gain cultural understanding of the clinical situation.^27^ Emotions can be as deceptive as statistics. That the clinician registers their own amazement does not mean the interpretation is accurate.

## Conclusions

Multicultural societies with increasingly complex health problems make the practice of culturally informed psychiatry urgent. This article has demonstrated that the encounter between the patient and the clinician is a crucial, yet underappreciated, position from which the clinician might develop a cultural understanding. The cultural dimension of the clinician–patient relationship must be explored in actual situations through the clinician's self-reflexive focus on amazement and questions such as, ‘How do I respond to situations where I become amazed?’, ‘Is it possible for me to develop understanding rather than withdrawal?’

The clinician is always formed by social and cultural contexts and is never culturally neutral. Moreover, the clinician has a clinical responsibility to make explicit his or her own assumptions, premises and categories in relation to patients to prevent misunderstandings and misdiagnoses. Culturally informed psychiatry cannot be defined once and for all; it is not a quick-fix technique or manual. It is rather the continuous development of a professional attitude, perceiving all human beings, including the clinician, as cultural bearers and cultural learners.^28^ The clinician should take seriously their own amazement as a point of entry to this attitude. However, to develop amazement as a clinician to strengthen culturally informed psychiatry does not lead to an easy resolution of a client's problem. A whole new series of questions arises, and we need culturally reflexive psychiatrists to deal with these questions.
